# Identification and Characterization of HTLV-1 HBZ Post-Translational Modifications

**DOI:** 10.1371/journal.pone.0112762

**Published:** 2014-11-12

**Authors:** Nathan Dissinger, Nikoloz Shkriabai, Sonja Hess, Jacob Al-Saleem, Mamuka Kvaratskhelia, Patrick L. Green

**Affiliations:** 1 Center for Retrovirus Research, The Ohio State University, Columbus, Ohio, United States of America; 2 Department of Veterinary Biosciences, The Ohio State University, Columbus, Ohio, United States of America; 3 Comprehensive Cancer Center and Solove Research Institute, Columbus, Ohio, United States of America; 4 Department of Molecular Virology, Immunology, and Medical Genetics, The Ohio State University, Columbus, Ohio, United States of America; 5 College of Pharmacy, The Ohio State University, Columbus Ohio, United States of America; 6 Proteome Exploration Laboratory, Beckman Institute, California Institute of Technology, Pasadena, California, United States of America; University of Hong Kong, Hong Kong

## Abstract

Human T-cell leukemia virus type-1 (HTLV-1) is estimated to infect 15–25 million people worldwide, with several areas including southern Japan and the Caribbean basin being endemic. The virus is the etiological agent of debilitating and fatal diseases, for which there is currently no long-term cure. In the majority of cases of leukemia caused by HTLV-1, only a single viral gene, *hbz*, and its cognate protein, HBZ, are expressed and their importance is increasingly being recognized in the development of HTLV-1-associated disease. We hypothesized that HBZ, like other HTLV-1 proteins, has properties and functions regulated by post-translational modifications (PTMs) that affect specific signaling pathways important for disease development. To date, PTM of HBZ has not been described. We used an affinity-tagged protein and mass spectrometry method to identify seven modifications of HBZ for the first time. We examined how these PTMs affected the ability of HBZ to modulate several pathways, as measured using luciferase reporter assays. Herein, we report that none of the identified PTMs affected HBZ stability or its regulation of tested pathways.

## Introduction

Human T-cell leukemia virus type 1 (HTLV-1) was the first human retrovirus discovered to be associated with diseases [1], [2] including the aggressive CD4^+^ T-cell malignancy, adult T-cell leukemia (ATL) [3], as well as the neurodegenerative disease HTLV-1-associated myelopathy/tropical spastic paraparesis, and other inflammatory diseases [4]. HTLV-1 encodes the structural and enzymatic proteins, Gag, Pol, and Env, as well as the regulatory proteins, Tax and Rex. The virus also encodes accessory proteins that are required for efficient infection and persistence *in vivo*, but are dispensable for T-cell immortalization *in vitro* [5]. The accessory/regulatory protein HBZ is unique in that it is the only viral protein encoded by the minus strand of the proviral genome while the rest of the viral proteins are encoded by the plus strand [6]–[8]. HBZ is expressed in all HTLV-1 cell lines and cases of ATL; in fact, in 60% of those ATL cases, *hbz* is typically the only viral gene expressed (eg. no Tax) [9]. This finding is attributed to deletion or hyper-methylation-silencing of the promoter in the 5′ LTR or a non-functional mutation in the Tax transactivator, which significantly disrupts plus-strand transcription [9], [10].

When HBZ was discovered, it was first shown to repress Tax transactivation of the viral promoter [7], [11], [12]. Since then, other functions have been reported such as modulation of the AP-1 [13]–[17] and the classical NF-κB signaling pathways [18], [19]. More recent studies have shown that HBZ may regulate the cell-mediated immune response to the virus infection [20], [21]. There also is growing evidence that HBZ is important in the oncogenic process since it plays a role in driving infected cell proliferation [22]–[24], increasing hTERT transcription [25], [26], and inhibiting apoptosis [20], [27].

Post-translational modifications (PTMs) are chemical modifications added to proteins that can alter many aspects of a protein, including conformation, localization, and activity. This common mechanism of cellular regulation is utilized by several pathogens, including HTLV-1, to alter the expression of their own proteins. Tax contains several PTMs, for example, phosphorylation of Tax both stabilizes the protein [28] and inhibits its activity [29]. In addition, a phosphorylation site is required for the addition of an acetyl group that activates Tax to enhance NF-κB and induce transformation [30], [31]. Furthermore, our lab has shown phosphorylation to be vital for the regulation of Rex function [32].

There currently are no published data about whether HBZ is post-translationally modified; however, it is known that HBZ interacts with acetyl-transferases [12], [33]. Therefore, we hypothesized that HBZ, like Tax and Rex, would contain PTMs that regulate important functions. In this study, we purified an affinity-tagged-HBZ protein and analyzed this protein by LC-MS/MS. A high percentage of the protein, including the majority of the key leucine-zipper domain at the C-terminus, was covered in this analysis. This approach identified 7 modifications, which were further characterized by mutational analysis to determine if they regulated known HBZ functions.

## Materials and Methods

### Cells

293T cells were maintained in Dulbecco's modified Eagle's medium and Jurkat T-cells were maintained in RPMI medium at 37°C in a humidified atmosphere of 5% CO_2_ and air. Media was supplemented with 10% fetal bovine serum (FBS), 2 mM glutamine, penicillin (100 U/ml), and streptomycin (100 µg/ml). Cells were originally obtained from ATCC.

### Plasmids

To generate the Flag-6xHis-HBZ construct, the HBZ cDNA was inserted downstream of an N-terminal Flag-6xHis affinity tag and expression was driven by a CMV promoter. Amino acid exchanges were made using the QuickChange site-directed mutagenesis kit (Stratagene, La Jolla, CA). All mutations were confirmed by DNA sequencing and expression was verified by transfection and Western blot analysis. The pCMV-c-Jun and pLG4-10-6xAP-1-Luc plasmids were graciously provided by Dr. John C. McDermott of York University. The p65 expression plasmid and κB-Luc plasmid were a kind gift from Dr. Dean Ballard of Vanderbilt University. The IRF-1 expression plasmid and IRF-1 luciferase reporter plasmid were graciously provided by Dr. John Yim of the Beckman Research Institute.

### Protein Purification

293T cells were plated in six 100 mm dishes, three per condition, and each plate was transfected with 10 µg of empty vector or Flag-6xHis-HBZ plasmid using lipofectamine (Invitrogen, Carlsbad, CA). Twenty-four hours post-transfection, cells were collected, combined, washed in cold 1x PBS, and lysed following the FLAG fusion protein immunoprecipitation and SDS-PAGE buffer elution protocols of the FLAG M Purification Kit (Sigma Aldrich, St. Louis, MO). Samples were loaded on a large 12% SDS-PA gel and electrophoresed for 3 hours at 55 mA. The gel was washed with Millipore water and stained using GelCode Blue Stain (Thermo Scientific, Rockford, IL). The HBZ band was excised from the gel for further proteomic analysis.

### Mass Spectrometry and Proteomic Analysis

LC-MS/MS analysis was performed as described previously [34] with following modifications. HBZ excised gel slices were cut into small pieces (2–3 mm cubes) and incubated on a shaker overnight in 50% acetonitrile to distain gel pieces from Coomassie dye. Samples were reduced with 7.5 mM DTT in 75 mM ammonium bicarbonate solution at 50°C for 30 min, after which DTT was removed and the protein was alkylated with 40 mM iodoacetamide in 75 mM ammonium bicarbonate solution for 20 min at room temperature in dark. The gel pieces were washed with acetonitrile, desiccated in a speed-vac. Aliquots were, subjected to in-gel proteolysis using the following endoproteinases (5 ng/µl): i) sequencing grade modified trypsin (Promega); ii) sequencing grade chymotrypsin (Roche); iii) sequencing grade endoproteinase Asp-N, (Roche) and iv) Trypsin/Asp-N combination. The resulting peptides were extracted in 100 µl of acetonitrile by vortexing for 10 min. The solution was transferred to new small microcentrifuge tubes and desiccated in a speed-vac. Dryed samples were resuspended in 6 µl buffer A (2% acetonitrile, 0.2% formic acid,) and 5 µl were separated on a 15 cm×0.075 mm fused silica capillary column packed with reversed phase 3 µm ReproSil-Pur C_18AQ_ resin (Dr. Maisch GmbH, Ammerbuch-Entringen, Germany) using a nano EASY HPLC. Peptides were eluted over 50 min by applying a 0–30% linear gradient of buffer B (80% acetonitrile, 19.8% water and 0.2% formic acid) at a flow rate of 350 nL/min. The Orbitrap (Thermo Fischer Scientific, San Jose, CA) was run in data dependent mode with 10 data-dependent scan events for each full MS scan. Normalized collision energy was set at 35, activation Q was 0.250. AGC target for MS was 1×10^6^ and AGC target for MS/MS was 5×10^4^. Dynamic exclusion was set to 60 s and early expiration was disabled. Sequence analysis was performed with MASCOT (Matrix Sciences, London GB) software using an indexed human subset database of Swissprot, supplemented with HTLV, 263 contaminants and 114960 decoy sequences.

### Reporter Assays

Each functional reporter assay had its own set of conditions for plasmid concentrations. In brief, 293 T cells were seeded in 6-well plates at 2×10^5^ cells per well. Twenty-four hours post-plating, cells were transfected with 10 or 20 ng of renilla-TK, a luciferase reporter, an expression plasmid of a specific transcription factor, and an HBZ expression plasmid at one of two concentrations (1∶5 or 1∶10 ratio). Empty vector was added to make the total DNA concentration equal among all transfections. Transfections were performed using Lipofectamine (Invitrogen, Carlsbad, CA). Twenty-four hours post-transfection, cells were collected and analyzed using a dual luciferase assay kit (Promega, Madison, WI). Levels of firefly luciferase and renillia luciferase were measured using a Packard LumiCount luminometer. Each experiment was performed three independent times in duplicates. Jurkat T-cells were plated in 6-well dishes at 3.5×10^5^ cells per well. Twenty-four hours post-plating, cells were transfected using TransFectin lipid reagent (Bio-Rad Laboratories, Hercules, CA) by following the manufacturer's guidelines. Forty hours post-transfection, cells lysates were collected and analyzed as described above.

### Western Blot Analysis

Transfected cells were lysed in 1x Passive Lysis Buffer (Promega, Madison, WI) with protease inhibitor cocktail (Roche, Mannheim, Germany). Protein concentrations were measured using a Nanodrop spectrophotometer (Thermo Fisher Scientific, Waltham, MA). SDS dye (6x solution) was added to the lysates and samples were boiled for 10 min. Twenty micrograms of protein were resolved by SDS-PAGE and transferred to nitrocellulose membranes. Blots were probed with a rabbit polyclonal anti-HBZ antiserum (1∶1000), a mouse anti-Flag M2 antibody (1∶5000) (Sigma Aldrich, St. Louis, MO), or mouse anti-Actin (1∶10000) according to standard procedures. Secondary antibodies used included goat anti-rabbit and goat anti-mouse conjugated with horseradish peroxidase (Santa Cruz Biotechnology, Santa Cruz, CA) at a dilution of 1∶2000. Blots were developed using Immunocruz luminol reagent (Santa Cruz Biotechnology) and imaged using the Fuji LAS 4000 imaging system (GE Healthcare Life Sciences, Piscataway, NJ). Densitometry was measured using Multi Gauge version 3.0 software (Fujifilm, Tokyo, Japan).

## Results

### Prediction of HBZ PTMs

There are currently no reports on whether HBZ is post-translationally modified, but it is well known that PTMs can play a major role in the properties and functions of proteins. Two major types of modification are phosphorylation and acetylation. These modifications are reversible and are used to modify the activity of many transcription factors [35]. Using the online phosphorylation prediction tool, NetPhos 2.0 Server [36], we found 6 potential phosphorylation sites on HBZ (Table 1). We also used the online tool PAIL [37] for acetylation prediction (Table 2) that predicted 16 acetylated lysines. These data suggested that HBZ was likely modified by the cell. Instead of mutating all predicted modified residues, we first set out to identify modified residues by performing mass spectrometry (MS). This approach would allow us to identify both phosphorylation and acetylation added to HBZ within a eukaryotic cell in a single assay. This analysis was dependent on the production and purification of substantial quantities of HBZ protein.

**Table 1:** Predicted and Identified Phosphorylation Sites of HBZ.

Predicted Phosphorylation Site	Score	Threshold	Times Modified/Times Detected	% Modified
S29	0.974	0.5	0/5	0.00%
S49	0.975	0.5	1/27	3.70%
S54	0.965	0.5	0/15	0.00%
T73	0.991	0.5	0/5	0.00%
S150	0.993	0.5	N.D.	N/A
S174	0.929	0.5	0/87	0.00%

N.D. means Not Detected. N/A means Not Applicable.

**Table 2:** Predicted and Identified Acetylation Sites of HBZ.

Predicted Acetylation Site	Score	Threshold	Times Modified/Times Detected	% Modified
K66	2.09	0.5	39/39	100.00%
K84	2.64	0.5	N.D.	N/A
K86	1.65	0.5	N.D.	N/A
K88	2.42	0.5	N.D.	N/A
K89	1.96	0.5	N.D.	N/A
K93	1.47	0.5	0/2	0.00%
K106	0.79	0.5	N.D.	N/A
K110	1.37	0.5	N.D.	N/A
K119	1.51	0.5	N.D.	N/A
K120	2.43	0.5	0/1	0.00%
K128	0.75	0.5	N.D.	N/A
K145	1.6	0.5	N.D.	N/A
K147	1.89	0.5	N.D.	N/A
K153	1.26	0.5	N.D.	N/A
K155	1.13	0.5	2/71	2.82%
K181	0.67	0.5	0/73	0.00%

N.D. means Not Detected. N/A means Not Applicable.

### Function and purification of Flag-6xHis-HBZ

**Figure 1:**
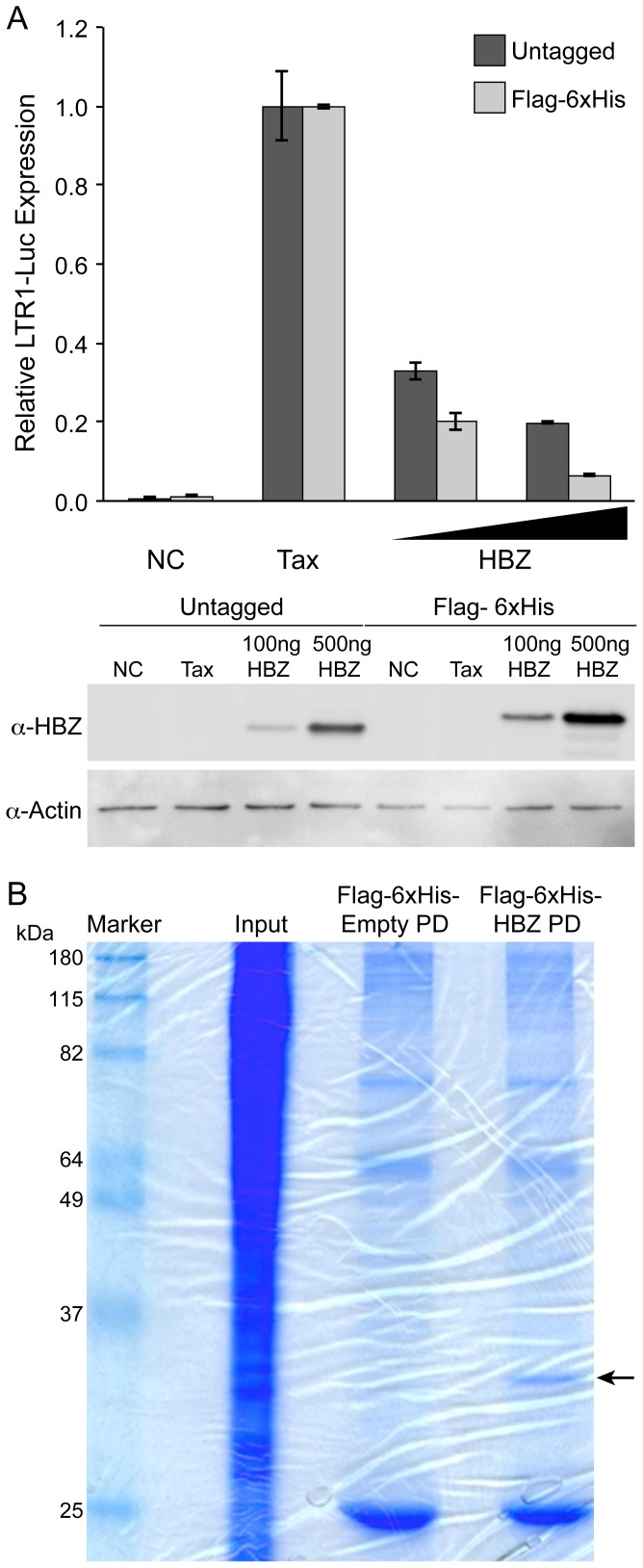
Flag-6xHis-tagged HBZ represses Tax transactivation and can be purified. (A) The functional activity of Flag-6xHis-HBZ was determined by measuring its ability to repress Tax trans-activation of a LTR1-luc reporter in 293T cells. Cells were transfected with S-tag Tax (200 ng), LTR-1 luciferase reporter (100 ng) renilla-TK (20 ng) and a titration of HBZ constructs (100 and 500 ng.) Values represent mean measurements relative to Tax expression alone. Error bars represent standard deviation. Western blots were performed on 20 ug total cell lysate and probed with rabbit HBZ-specific antisera. Actin was used as a loading control. (B) Flag-6xHis-HBZ was purified from lysates of transfected 293T cells using agarose beads conjugated with anti-Flag antibody. Bands were resolved by SDS-PAGE and the gel was stained with GelCode Blue.

Varying amounts of the Flag-6xHis-HBZ construct were transfected into 293T cells along with a Tax expression plasmid and the HTLV-1-LTR-Luciferase reporter plasmid (Figure 1A). As expected, Flag-6xHis-HBZ was able to repress Tax transactivation in a dose-dependent manner similarly to untagged, wild-type HBZ. We next verified that we would be able to adequately purify HBZ for mass spectrometry analysis. Using components of Sigma's FLAG M Purification kit, lysates from HBZ-transfected 293T cells were collected and HBZ was purified using agarose beads conjugated with mouse anti-Flag antibody. SDS-PAGE and GelCode Blue visualization revealed a specific band correlating to tagged HBZ that could be processed for mass spectrometry (Figure 1B).

### Identification of PTMs

**Figure 2:**
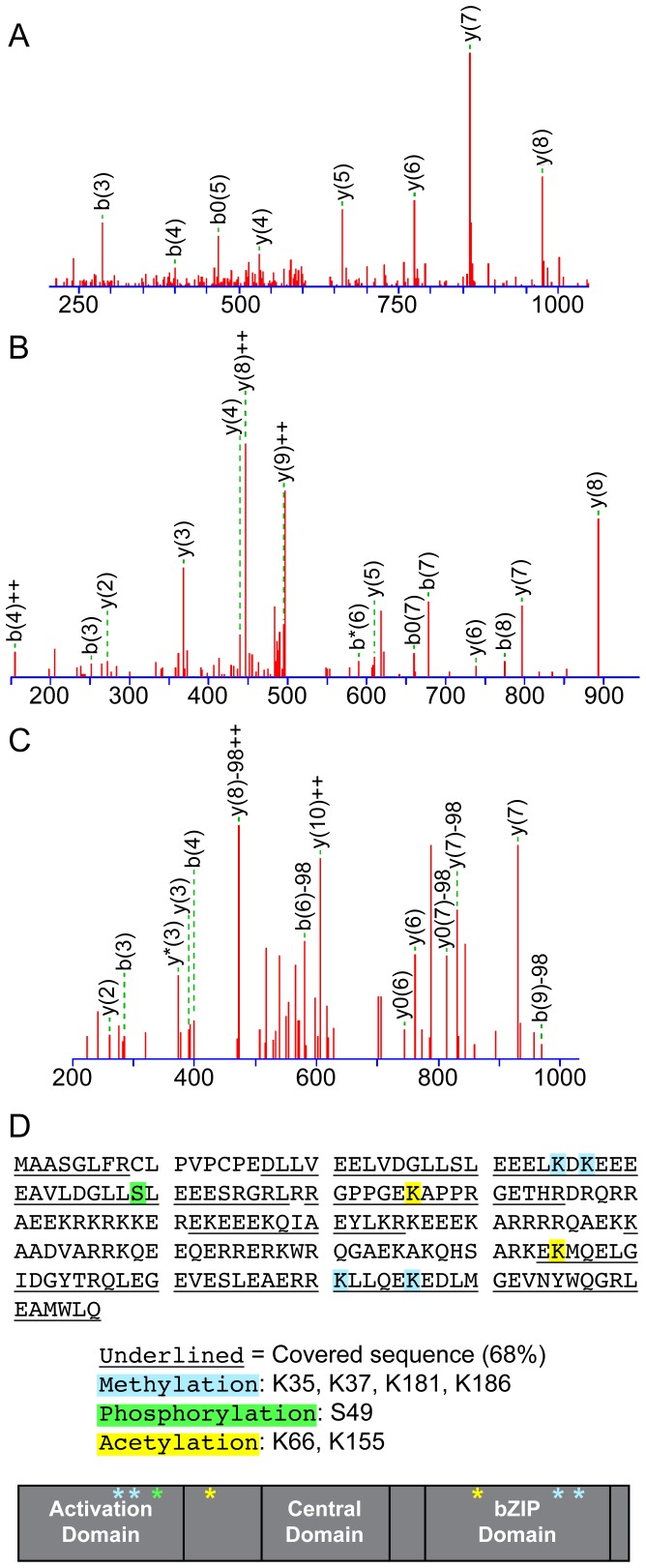
MS based analysis of PTMs in HBZ. (A) MS/MS fragmentation of HBZ peptide DGLLSLEEELK*, where Lys is methylated. (B) MS/MS Fragmentation of HBZ peptide GPPGEK*APPR, where Lys is acetylated. (C) MS/MS Fragmentation of HBZ peptide DGLLS*LEEESR, where Ser is phosphorylated. (D) Summary of MS results. The HBZ sequence is provided, with covered sequences underlined. PTMs found are highlighted in the sequence and marked relative to the three domains of HBZ in a cartoon.

Multiple runs of LC-MS/MS were performed with protein digestion schemes described in the Materials and Methods section. Overall, we were able to obtain 68% coverage of the amino acid sequence, including the majority of the key leucine-zipper functional domain and identified several PTMs (Figure 2). We detected phosphorylation on S49, acetylation on K66 and K155, and methylation on K35, K37, K181 and K186 (Figure 2 and Tables 1–3). The majority of these modifications occur in the important protein-protein interaction domains of HBZ. Of the predicted phosphorylation sites, we covered 5 of the 6 predicted sites (Table 1), and 5 of the 16 predicted acetylation sites (Table 2). We compared MS spectrum counts for modified peptides with their unmodified counterparts, which allowed semiquantitative analysis for the frequency of modifications (Tables 1–3). Our data suggest that the phosphorylation of HBZ is an infrequent occurrence since S49 showed limited phosphorylation. The addition of an acetyl group to K155 also seems to be a rare event, being detected approximately 3% of the time. Of the discovered methylations, our data indicate that only K35 is methylated with some consistency. Furthermore, we provide evidence that K66 is constitutively acetylated, neutralizing the positive charge of this amino acid. All these identified modifications are novel and, we hypothesized, could regulate the properties and/or functions of HBZ.

**Table 3:** Detected Methylation Sites of HBZ.

Methylated Residue	Times Modified/Times Detected	% Modified
K35	3/10	30.00%
K37	1/6	16.67%
K181	3/73	4.11%
K186	1/80	1.25%

### PTMs do not affect HBZ steady-state levels

In the current study, we decided to examine the roles of phosphorylation and acetylation individually by mutating modified amino acids to mimetic (S→D and K→Q, respectively) and inhibitory (S→A and K→R, respectively) residues for each PTM. We also created a phospho-, acetyl-mimetic mutant (PhAc-mim: S49D-K66Q-K155Q), and a phospho-, acetyl-inhibitory mutant (PhAc-inh: S49A-K66R-K155R) for the discovered modified sites to investigate if they act in concert. The approach of having mimetic and inhibitory mutants allowed us to compare each mutant to the wild-type protein and the paired residue mutation. It also is important to examine both the mimetic and inhibitory mutations as both phosphorylation and acetylation can positively or negatively regulate the functions of proteins. We decided not to focus on the discovered methylation sites currently since all methylated states were found in less than half the cases of detected peptides and we cannot create a methylated lysine mimetic mutation. If future studies identify methyltransferases that interact with HBZ, it would be interesting to see if over-expression of these enzymes modifies these residues of HBZ and are important for HBZ function.

**Figure 3:**
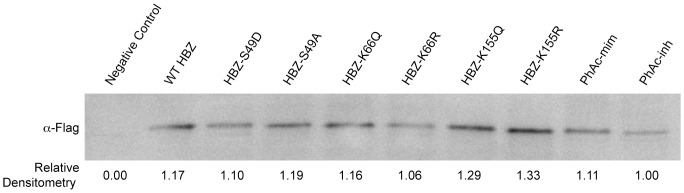
Steady-state levels of HBZ protein is not altered by identified PTMs. Lysates were collected from transfected 293 T cells and were resolved by SDS-PAGE. The amount of lysate loaded was normalized to measured renilla-TK values. The blot was probed with mouse anti-Flag M2 (1∶5000). Densitometries were measured and the ratios are reported under the blot.

After the creation of the mutant forms of HBZ in the Flag-6xHis vector, Western blot analysis was performed to examine if any of the modifications affected the steady-state levels of the protein. We hypothesized that acetylation of K66 would be important for protein stability as it was found to be constitutively modified. However, probing for the affinity-tag showed that none of the modifications affected the steady state level of the protein (Figure 3).

### PTMs do not affect inhibition of viral regulatory proteins

**Figure 4:**
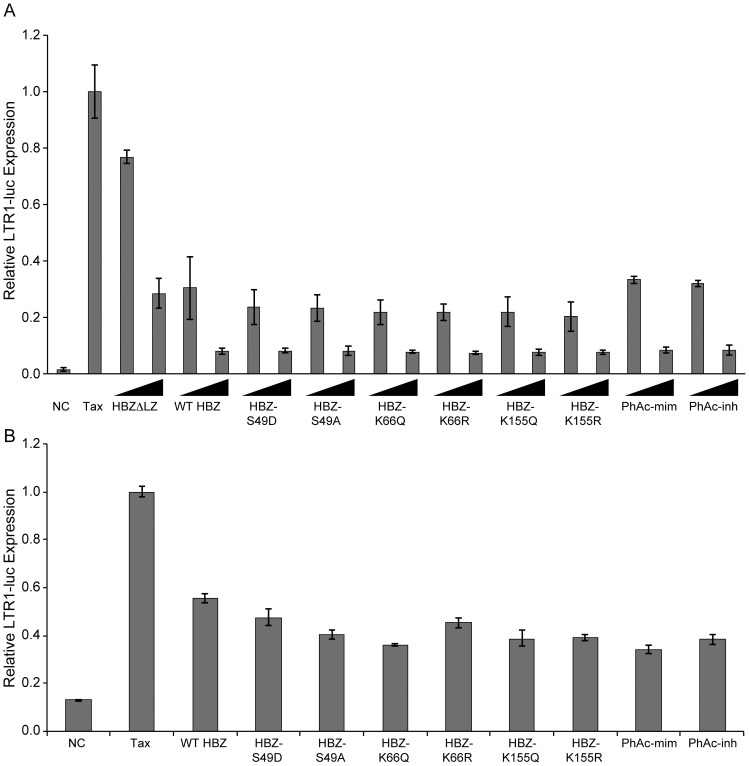
Phosphorylation and acetylation do not alter the ability of HBZ to repress Tax transactivation of the LTR promoter. (A) 293 T cells were transfected with LTR-1-luc reporter (100 ng), renilla-TK (20 ng), S-tagged Tax (200 ng) and a titration of HBZ mutants (100 ng and 500 ng). DNA amounts were normalized with empty Flag-6xHis plasmid. Twenty-four hours post-transfection, lysates were collected and luciferase levels were measured. (B) Jurkat T-cells were transfected with LTR-1-luc reporter (300 ng), renilla-TK (100 ng), S-tagged Tax (600 ng) and HBZ mutants (1500 ng). Forty hours post-transfection, lysates were collected and luciferase levels were measured. Error bars represent standard deviations.

HBZ inhibits Tax-transactivation of the LTR promoter by binding to the co-activators CREB and p300 [11], [12] and by up-regulating the ubiquitin E3 ligase PDLIM2, which targets Tax for degradation [38]. To examine if the phosphorylation and acetylation of HBZ were important for this function, 293 T cells were transfected with an HTLV-1 LTR-luciferase reporter along with Tax and titrating amounts of wild-type HBZ, the PTM mutants, and a ΔLZ mutant (previously shown to repress Tax-transactivation to a lesser extent than wild-type) [11], [12] (Figure 4A). As expected, wild-type HBZ and, to a lesser degree, HBZΔLZ, were able to repress Tax trans-activation of the HTLV-1 LTR promoter. We observed that all PTM mutants tested were able to inhibit Tax activity to a similar degree as wild-type HBZ and found no significant difference between paired mutants. It should be noted that this result for the S49 mutants was not unexpected because these mutations were reported previously to bind to p300, inhibiting it from interacting with Tax [12]. To examine if the PTMs affected HBZ's functions within a T-cell, the luciferase assay was repeated in Jurkat T-cells (Figure 4B). Results in Jurkat T-cells were similar to 293 T cells; all the tested PTM mutants functioned similarly to wild-type HBZ and repressed Tax-transactivation of the HTLV-1 LTR promoter. These data suggest that the tested PTMs do not affect the ability of HBZ to modulate Tax activity. More recently, it has been reported that HBZ modestly repressed Rex function in a dose-dependent manner in HeLa cells [39]. We confirmed this repression of Rex activity by wild-type HBZ and that all of the PTM mutants functioned similarly to wild-type HBZ (data not shown). These data suggest that the tested PTMs do not affect the ability of HBZ to modulate Rex activity.

### PTMs of HBZ do not affect p65 or c-Jun transcriptional activity

**Figure 5:**
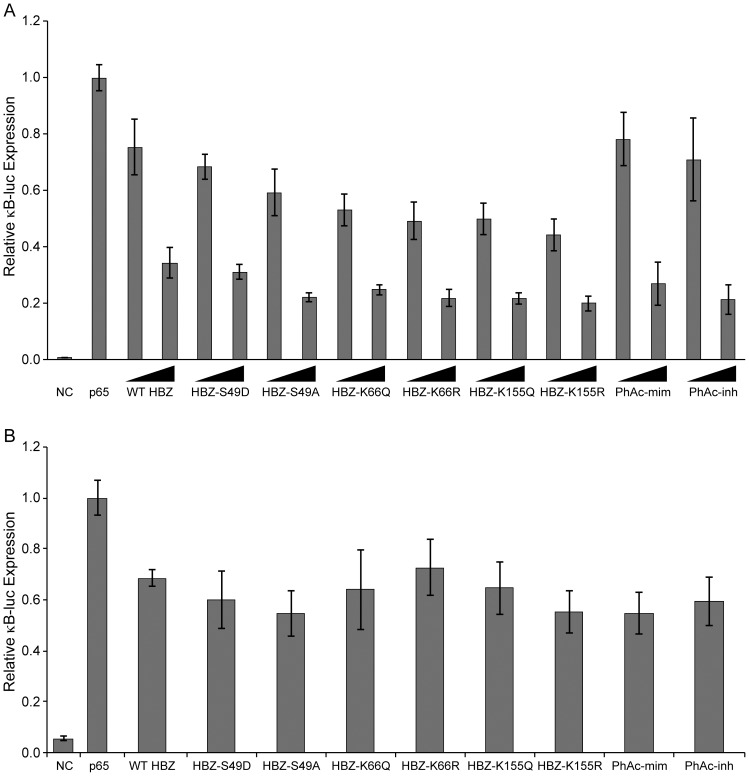
Phosphorylation and acetylation do not affect the ability of HBZ to repress p65-mediated transcription. (A) 293 T cells were transfected with κB-luc reporter (100 ng), renilla-TK (20 ng), p65 expression plasmid (50 ng) and a titration of HBZ constructs (100 ng and 500 ng). DNA amounts were normalized with empty Flag-6xHis plasmid. Lysates were collected 24 hours post-transfection and luciferase levels were measured. (B) Jurkat T-cells were transfected with κB-luc reporter (200 ng), renilla-TK (100 ng), p65 expression plasmid (100 ng) and HBZ mutants (1000 ng). Lysates were collected 40 hours post-transfection and luciferase levels were measured. Error bars represent standard deviations.

It previously was reported that HBZ represses the classical NF-κB pathway by inhibiting the DNA-binding of p65 and inducing p65 degradation [18]. This finding was important because this binding stopped cells from entering Tax-induced senescence [19]. We used a reporter assay to examine if the discovered PTMs affected the ability of HBZ to repress p65 in both 293 T cells and Jurkat T-cells (Figure 5A and 5B). These data demonstrated that the PTMs of HBZ, individually and in combination with each other, did not affect HBZ's ability to repress p65 transcriptional activity in either cell type.

**Figure 6:**
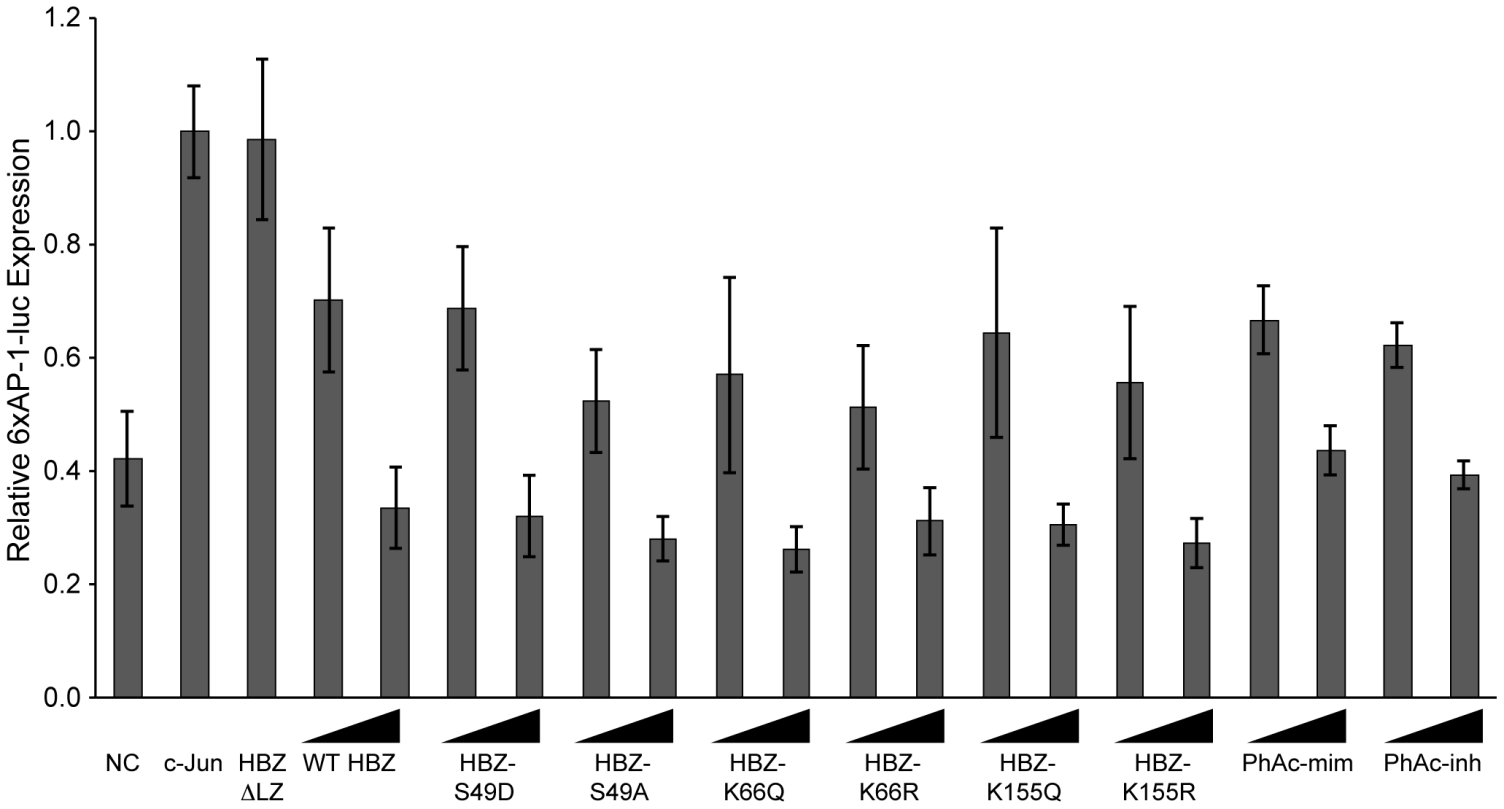
Phosphorylation and acetylation do not affect the ability of HBZ to inhibit c-Jun-mediated transcription. 293 T cells were transfected with a 6xAP-1-luc reporter (50 ng), renilla-TK (10 ng), pCMV-c-Jun (25 ng) and a titration of HBZ constructs (10 ng and 100 ng). DNA amounts were normalized with empty Flag-6xHis plasmid. Lysates were collected 24 hours post-transfection and luciferase levels were measured. Error bars represent standard deviations.

We next examined how the PTMs affect the ability of HBZ to repress c-Jun transcriptional activity because HBZ and APH-2, the HBZ counterpart in non-pathogenic HTLV-2, differentially regulate this cellular pathway [13], [40]. 293 T cells were transfected with a 6xAP-1-luciferase construct along with pCMV-c-Jun and titrating amounts of HBZ. Because HBZ interacts with c-Jun through its leucine zipper domain [13], we also included the HBZΔLZ mutant (Figure 6). Our results show WT HBZ was able to repress c-Jun-mediated transcription and the HBZΔLZ mutant was unable to repress c-Jun. All PTM mutants acted in a manner similar to WT HBZ, indicating none of the PTMs affected the interaction of HBZ with c-Jun. Taken together, our data indicate that the phosphorylation and acetylation state at these residues are not important for the ability of HBZ to modulate the classical NF-κB and AP-1 pathways.

### Repression of IRF-1 is not dependent on HBZ PTMs

**Figure 7:**
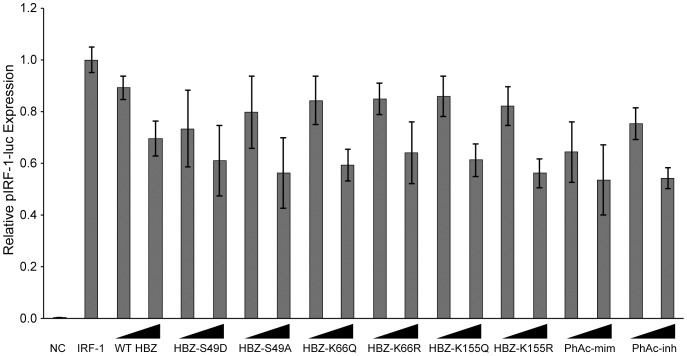
The ability of HBZ to repress IRF-1 activity is not affected by the identified PTMs. 293 T cells were transfected with pIRF-1-luc (50 ng), renilla-TK (20 ng), IRF-1 expression plasmid (100 ng) and a titration of HBZ constructs (50 ng and 500 ng). DNA amounts were normalized with empty Flag-6xHis plasmid. Lysates were collected 24 hours post-transfection and luciferase levels were measured. Error bars represent standard deviations.

After testing whether PTMs regulate the ability of HBZ to repress viral expression and growth pathways, we next turned our attention to a component of the innate immune system. Interferon (IFN) regulatory factors (IRFs) are key components of the immune system as they control interferon production and development of immune cells, but they also play a role in regulating oncogenesis [41]. IRF-1 induces the expression of type-I IFN and acts as a tumor suppressor by inducing apoptosis [42]. Clinical data have shown that IRF-1 expression is lost in many cases of leukemia [43]. Since HBZ is typically the only HTLV-1 protein expressed in cases of ATL, Mukai et al investigated if HBZ and IRF-1 interacted [20]. They discovered that the N-terminus of HBZ was important for binding IRF-1 and repressing its activity. We performed a reporter assay to assess whether the PTMs of HBZ regulated the repression of IRF-1 transcriptional activity (Figure 7). All PTM mutants were able to repress IRF-1 in a dose-dependent manner and there were no significant differences between paired mutations. These data suggest that PTMs are not involved in the regulation of IRF-1 activity by HBZ.

## Discussion

Our present research is the first to report PTMs of HBZ and to define the potential role of these PTMs in the known functions of HBZ. Using online prediction tools, we found 6 potential sites of phosphorylation and 16 sites of acetylation. Our MS data covered 68% of the amino acids of HBZ, including 5 of the 6 potential phosphorylation sites and 5 of the 16 acetylation sites. In total, 7 modifications were identified: 1 phosphorylation, 2 acetylations, and 4 methylations. Three of these modifications occur in the N-terminal activation domain, one in between the activation domain and central domain, and the final three occur in the leucine zipper domain. Only acetylation of K66 occurred at a high frequency, with the 6 other modifications occurring at low frequency. The negative predicted sites that were covered cannot be fully ruled out as being modified, but we are confident that the modifications do not occur at a high frequency. Of the uncovered predicted sites, it would seem likely that acetylation would occur more frequently than phosphorylation because there are more sites available and HBZ is known to interact with acetyl-transferases.

We first examined the effect that these PTMs had on the steady state levels of the protein, but found no difference between samples and controls. We next tested how PTMs affect the ability of HBZ to repress Tax transactivation. Because none of the mutants acted differently than the wild-type at a low or high concentration, we are able to infer two aspects of the identified phosphorylation and acetylation: 1) they do not affect the interaction of HBZ with p300, and 2) they do not affect the interaction of HBZ with CREB. The cellular signaling pathways AP-1 and NF-κB, along with IRF-1-mediated transcription were examined. Although the modifications found were in domains that modulate the activity of the tested transcription factors, they did not play any role in the ability of HBZ to repress these selected pathways.

HBZ is known to interact with several proteins and affect various cellular pathways. While we could not identify any role for PTMs in the pathways examined, it remains possible that these PTMs have a function. Although the enzymes that add PTMs to their cognate proteins within the cell are not 100% specific for functionality, their promiscuity is still expected to be limited due to the importance of strict regulation and localization. The possibility that a combination of identified and unidentified PTMs may be necessary cannot be ruled out at this point. Furthermore, it is important to note that there could be unknown functions of HBZ that are regulated by these three PTMs. Future studies should focus on modifications that cannot be readily detected by MS such as SUMOylation [44], [45] as these have also been shown to be important for regulating protein functions.
